# Dr. Bliss’ Report of President Garfield’s Case

**Published:** 1881-10

**Authors:** 


DR. BLISS' REPORT OF PRESIDENT GARFIELD'S CASE.—As an apology to our readers for the delay in this issue of the INDEPENDENT PRACTITIONER, we call attention to the full report of the clinical history, treatment and autopsy in the case of President Garfield, published by Dr. Bliss in the *New York Medical Record* of October 8th. We reproduce from the *Record* the entire article, with all the original illustrations, together with a *Surgico-anatomical Study of the Wound* by Prof. Faneuil D. Weisse of this city. We are sure our readers will excuse the delay, since it has secured to them an early and complete history of a memorable case.

